# Digital Support for Healthier Eating Habits Among Patients With Type 2 Diabetes: Protocol for a Randomized Clinical Trial Within Primary Care (HAPPY Trial)

**DOI:** 10.2196/24422

**Published:** 2020-11-16

**Authors:** Ylva Trolle Lagerros, Anna Dahlgren, Linnea Sjöblom, Stephanie E Bonn

**Affiliations:** 1 Clinical Epidemiology Division Department of Medicine, Solna Karolinska Institutet Solna Sweden; 2 Center for Obesity Academic Specialist Center Stockholm Health Services Stockholm Sweden

**Keywords:** body composition, diabetes, dietary intake, HbA_1c_, metabolic health, mHealth, obesity, randomized clinical trial, serum lipids, smartphones

## Abstract

**Background:**

Despite the large impact that dietary habits have in the management of diabetes, few tools for supporting healthy eating habits are available for persons with diabetes.

**Objective:**

The aim of this randomized clinical trial is to evaluate the effect of a 12-week, mobile health (mHealth), app-based intervention promoting healthy eating habits among patients with type 2 diabetes.

**Methods:**

The HAPPY (Healthy eating using APP technologY) trial is a randomized clinical trial with two arms aiming to include 200 patients, 18 years of age or older, with type 2 diabetes. Both women and men are eligible for inclusion. Study participants are randomized 1:1 to an intervention group, where they are instructed to use a smartphone app promoting healthy eating, or to a control group, where they receive standard primary care only, for a period of 12 weeks. Each week a new topic (eg, vegetable intake) is introduced via the app. After an introduction text, the user is given a topic-related activity to perform (eg, eat one additional serving of vegetables per day during that week). The app records daily progress and sends automatic reminders or feedback to the user. Dietary intake, body composition, clinical variables, and biomarkers are measured at baseline and at 3- and 6-month follow-ups. An extensive web-based questionnaire comprising several validated questionnaires assessing a number of lifestyle factors is distributed via email at baseline and at 3-, 6-, and 12-month follow-ups; lifestyle factors include, for example, sleep, physical activity, eating behavior, and health-related quality of life. The effect of the intervention on dietary intake (primary outcome) and on glycated hemoglobin and blood lipid levels, body composition, blood pressure, other lifestyle factors, and overall health (secondary outcomes) will be assessed.

**Results:**

Data collection is ongoing. Recruitment of participants started in January 2019. Findings from the study are expected to be published by the end of 2021.

**Conclusions:**

Technology development provides new ways to promote and support long-term adherence to healthier eating habits. mHealth-based approaches allow for real-time interaction and the delivery of an intervention at any time. Further, focusing on overall diet allows the user to apply new knowledge to current eating patterns, creating an individualized approach. In this study, we evaluate the effect of using a new smartphone app promoting healthy eating habits on dietary intake, clinical markers, and lifestyle factors among patients with type 2 diabetes.

**Trial Registration:**

ClinicalTrials.gov NCT03784612; https://clinicaltrials.gov/ct2/show/NCT03784612

**International Registered Report Identifier (IRRID):**

DERR1-10.2196/24422

## Introduction

### Background

The prevalence of type 2 diabetes is increasing in Sweden, as well as in the rest of the world. Today, over 400 million adults have diabetes [1]. It has long been known that persons with type 2 diabetes have an increased risk of cardiovascular disease (CVD) and premature death, in large part due to an increased prevalence of risk factors for CVD (eg, obesity, dyslipidemia, and hypertension) [2].

More than 80% of patients with type 2 diabetes in Sweden are overweight or obese [3]; excess adiposity, in particular visceral obesity, as well as insulin resistance are strongly associated with an increased risk of both type 2 diabetes and CVD [4]. A healthy diet is the key factor in both prevention and management of type 2 diabetes. Patterns of vegan, vegetarian, and Mediterranean diets have been shown to improve glycemic control in patients with type 2 diabetes in randomized clinical trials [5]. An unhealthy lifestyle (eg, with poor dietary habits and physical inactivity), on the other hand, increases the risk of developing the disease, as well as the risk of complications [1]. In addition to CVD, complications can be serious and may include kidney failure, blindness, and amputation of lower extremities, and could lead to premature death.

Despite the large impact of dietary habits in the management of type 2 diabetes, few tools for supporting dietary changes and maintenance of a healthy diet are available. One strategy to improve health is regular visits to health clinics [6]. This may not always be a feasible strategy given the large number of patients in need of support. To meet both the needs of the patients and the capacity of the health care system, new strategies must be developed. Mobile health (mHealth) (ie, the use of mobile devices, including smartphones, to promote health) is one way to bridge the gap between what patients need and what health care can offer.

In Sweden, over 90% of adults own and use a smartphone [7], making an app-based intervention feasible for implementation in the general population of patients with type 2 diabetes. Technology-driven diabetes prevention programs (ie, utilizing, for example, text messages, email, automated phone calls, websites, etc) focusing on diet and/or physical activity have been evaluated in several intervention studies focusing on weight loss with promising results [8]. Michaelides et al [9] also showed that a fully mobile diabetes prevention program including a dietary component could facilitate weight loss and weight maintenance. Further, results from two recent reviews summarizing smartphone apps targeting diet have also shown promising results with regard to dietary intake in particular, although none of the apps had been developed for, or evaluated in, patients with type 2 diabetes specifically [10,11]. Nevertheless, among patients with type 2 diabetes, an automated web-based program to support healthy diet has been shown to improve dietary habits when assessed using a quality dietary score [12], and improvements in adherence to the Mediterranean diet and in diet quality overall have been shown after the use of a smartphone app during a period of 3 months [13]. Thus, mHealth strategies show potential as a tool to promote and support healthy eating habits, including among type 2 diabetes patients.

### Aim

We are conducting a randomized clinical trial called the HAPPY (Healthy eating using APP technologY) trial, which is a 12-week mHealth intervention (ie, includes the use of a smartphone app). The main aim of the trial is to evaluate its effect in promoting and improving healthy eating habits (primary outcome) and in improving levels of glycated hemoglobin (HbA_1c_), blood lipids, body composition, and blood pressure, as well as other lifestyle factors and overall health (secondary outcomes) in patients with type 2 diabetes. The aim of this paper is to describe the study design and methodology of the HAPPY trial.

### Hypothesis

Our hypothesis is that participants randomized into the intervention group, who will use the smartphone app, will have improved their dietary habits, cardiovascular risk factors, and other lifestyle factors after 12 weeks of active intervention compared to the control group, who will receive standard care. Further, we hypothesize that improved habits will be maintained after an additional 12 weeks of follow-up.

## Methods

### Study Design

The HAPPY trial is a randomized clinical trial with two arms. The research team behind the design of the smartphone app and the study includes nutritionists, epidemiologists, statisticians, and clinicians. The intervention is described according to the CONSORT (Consolidated Standards of Reporting Trials) statement [14] and the CONSORT-EHEALTH (Electronic and Mobile HEalth Applications and onLine TeleHealth) checklist developed specifically for eHealth and mHealth interventions [15].

Patients with type 2 diabetes who volunteer to participate are recruited from primary health care centers in the center and suburbs of Stockholm. Data collection is performed in collaboration with clinicians and nurses at the centers, where they give a brief introduction of the study to their patients. Information about the study is also available in the waiting rooms of the centers, and patients can contact study personnel directly if they are interested in participating. The included primary care centers differ in size, and the number of eligible patients can vary. Those interested in participating in the study are contacted by phone by study personnel and are given more information. Patients that fulfill inclusion criteria and agree to participate are sent an email with a link to the baseline questionnaire, including a web-based consent form. They are thereafter scheduled for a meeting with study personnel. During this meeting, participants sign an additional written informed consent form in order to verify that they fully understand the web-based information. Thereafter, baseline measurements are performed and patients are then randomized 1:1 to the intervention or control group. All participants are followed up after 3, 6, and 12 months.

**Figure 1:**
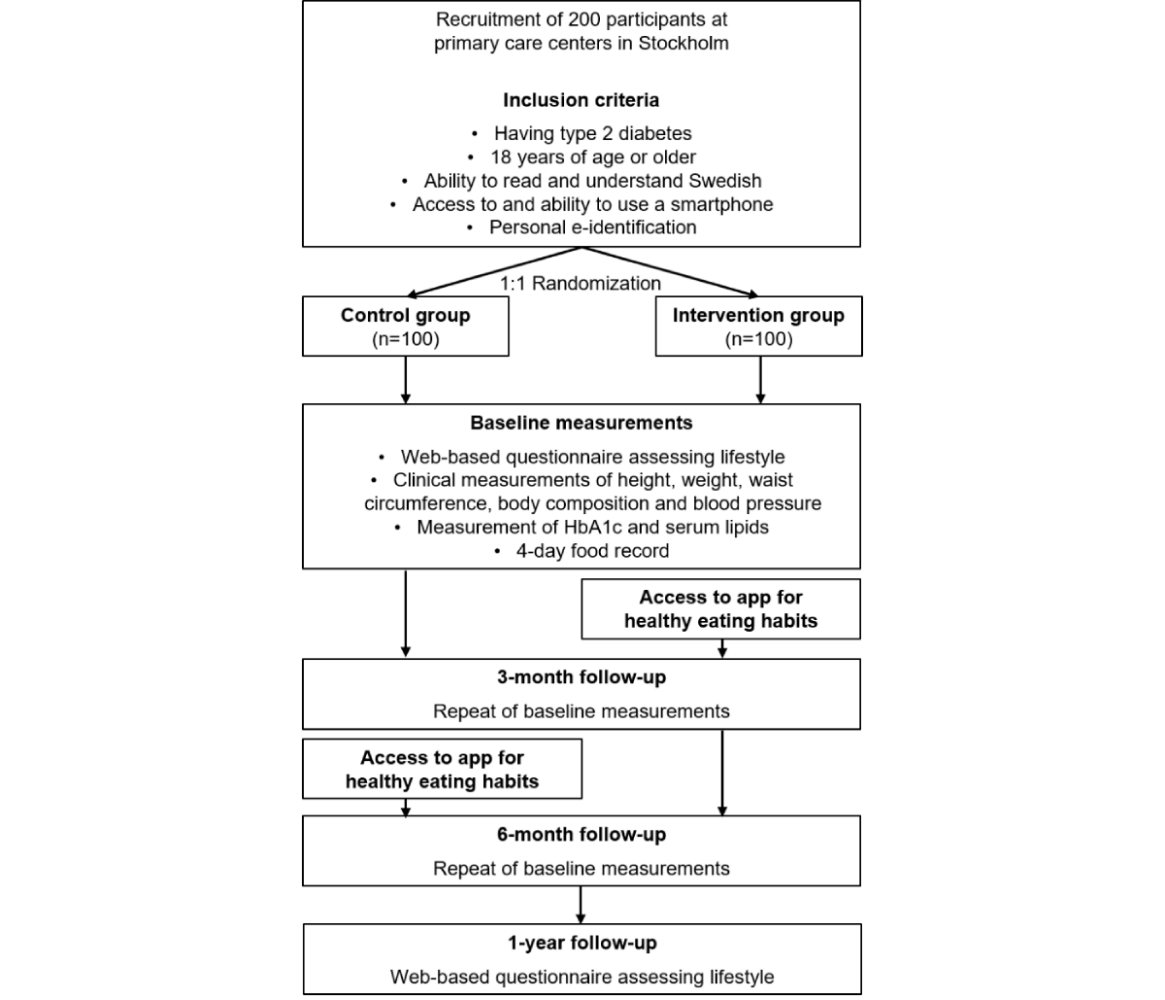
Study design of the randomized clinical intervention, the HAPPY trial, aiming to evaluate the use of an app-based program for healthy eating habits. HAPPY: Healthy eating using APP technologY; HbA_1c_: glycated hemoglobin.

Baseline and follow-up assessments at 3 and 6 months include an extensive web-based questionnaire including assessment of lifestyle factors; a 4-day food record; clinical measurements of height, weight, waist circumference, blood pressure, and body composition; and blood sampling for measurement of HbA_1c_ and serum lipids. After 12 months, participants are followed up with a final web-based questionnaire. All participants continue to receive usual care by their primary caregiver (ie, they visit their primary caregiver as planned as if they had not been part of the study). Participants randomized to the intervention group use the smartphone app during the 12 weeks of the active intervention from baseline, while participants in the control group will use the app during the 12 weeks after the first follow-up at 3 months. Participants are encouraged to use the app daily, but there is no requirement of how often participants should use the app. The study design is presented in Figure 1.

### Ethical Approval, Trial Registration, and Consent to Participate

The trial was approved by the ethics committee of the Regional Ethical Review Board, Stockholm, Sweden (2018/652-31; 2018/1094-32; 2018/2393-32). The trial was registered at ClinicalTrials.gov (NCT03784612). All study participants receive oral and written information about the study and give their written informed consent prior to study start.

### Inclusion and Exclusion Criteria

Inclusion criteria are having type 2 diabetes diagnosed by a physician; being 18 years of age or older; having the ability to read and understand Swedish; having access to, and the ability to use, a smartphone; having a personal e-identification (ie, a secure, digital citizen e-identification solution to enable personal and secure identification in the app); and giving informed consent for participation. Both women and men are eligible for inclusion. No specific exclusion criteria apply.

### Randomization and Blinding

Study participants are randomized to either the intervention group (ie, standard care and use of the smartphone app at study start) or the wait-listed control group (ie, standard care and use of the smartphone app after 3 months). Randomization is done by study personnel (AD and LS) at baseline at a 1:1 ratio using an allocation sequence list generated in Stata, version 14.0 (StataCorp LP). Women and men are randomized separately in blocks of 4 within each participating primary care center in order to assure an even distribution between the intervention and control groups. Due to the nature of the intervention, participants are not blinded to their allocation.

### Intervention

#### Development and Content of the HAPPY Smartphone App

The primary aim of the intervention is to achieve an improvement in dietary intake and subsequent improvements in clinical variables and lifestyle factors through the use of a smartphone app for healthy eating habits among patients with type 2 diabetes. The content of the HAPPY smartphone app and the healthy eating behavior program is built into an existing digital platform developed by FRISQ AB. The program with its contents has been developed by researchers with experience in behavior change interventions (eg, physical activity), nutritionists, and active clinicians within primary and specialist care. It is based on a theoretical framework including the health belief model and the stages of change model, as well as on social cognitive theory [16]. Several techniques for behavior change, including general information, goal-setting strategies, self-monitoring, and feedback on performance, are included in the program [17]. The specific features of the HAPPY smartphone app are described below. The app is available both for iOS (version 11.4 and higher) and Android (version 5.0 and higher).

#### The HAPPY Smartphone App

Each week the user will be introduced to a new topic on healthy eating habits (eg, vegetable intake). The specific topics for each of the 12 weeks of the active intervention are outlined in Table 1. Following a short written introduction to the current topic, the user is given an activity to perform (eg, to add one portion of vegetables per day during that week or replace sugar-sweetened beverages with water during a week). Daily progress is recorded in the app, and automatic reminders (eg, notifications if the user has not responded to an activity) or feedback on the activity (eg, “Thank you for participating in the activity”) will be given. All study participants receive the same introduction and activity to perform. Reminders and feedback depend on the actions taken by the user.

**Table 1:** Topics of the healthy eating program for each week of the intervention.

Week	Topic
1	Healthy food patterns
2	Vegetable intake
3	Regular eating habits
4	Sugar intake
5	More on vegetable intake
6	Slow and fast carbohydrates
7	Whole grains and fibers
8	Legumes
9	Saturated fat
10	Unsaturated fat
11	Salt intake
12	Beverages

**Figure 2:**
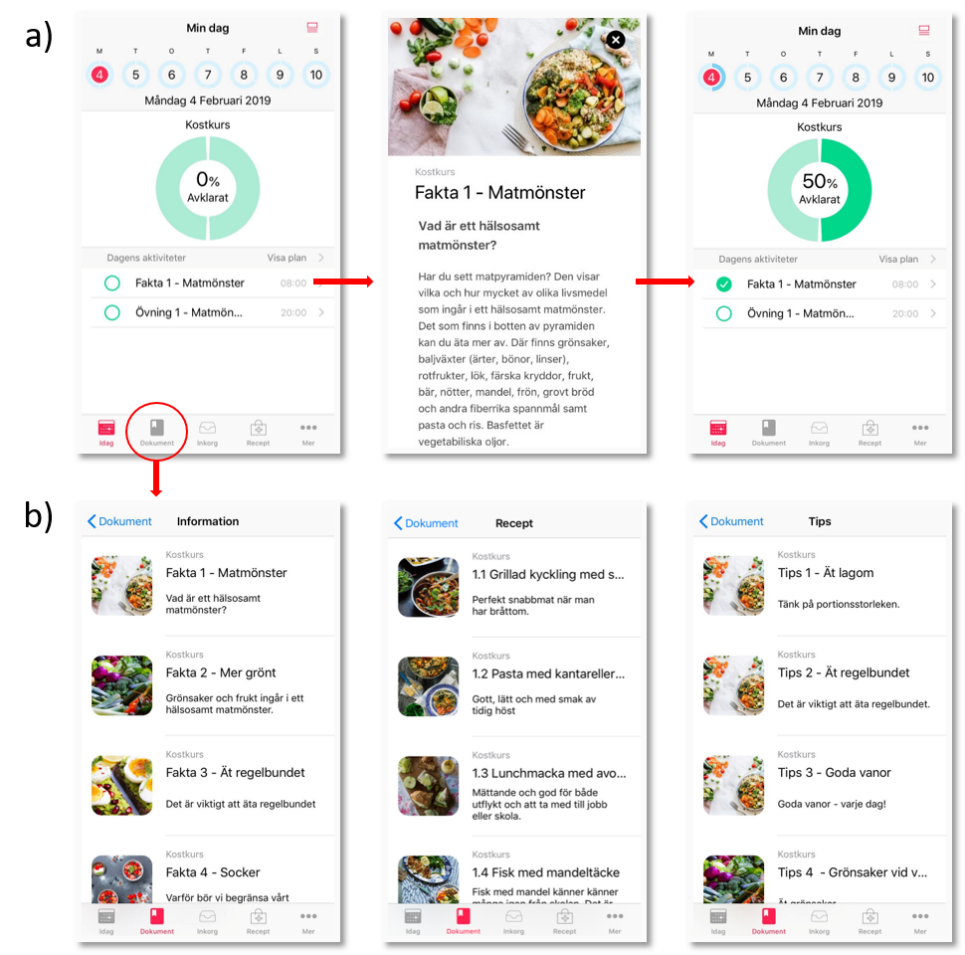
Examples of screenshots from the HAPPY smartphone app. a) Each day, the specific activities for that day are displayed to the user. Before completing an activity, 0% of the activities for that specific day have been performed, as indicated by the large circle. By touching the screen where an activity is listed, the user is taken to a new page showing the activity in question. When an activity has been completed, this is indicated in the large circle as well as to the left of the activity in the list below the circle. b) At the bottom of the first page in the app, the user can choose the document symbol to display information (left-hand image), recipes (middle image), or short facts and advice (right-hand image). The user can access the documents at any time; for example, they can return to reread information or find favorite recipes. HAPPY: Healthy eating using APP technologY.

Each topic (ie, each week) comprises information, recipes, short fun facts, or advice (ie, edutainment and an activity related to the topic). Every Monday, information and an activity that is linked to the theme of the week are available in the app. Recipes to inspire healthy cooking and facts or advice on healthy eating habits are introduced during the week on Tuesday, Wednesday, Friday, and Saturday. Every Thursday, the user receives a reminder of the activity, and at the end of the week, an evaluation about the activity is sent to the user. Each action (ie, reading the information text, recipes, short facts, or advice, and then performing the activity) is marked as read or completed in the app by the user. The status of an action in the app (ie, read vs not read or completed vs not completed) is also visible to study personnel. This information, together with information on each time a user has logged on to the app, is saved within the system. The structure of one week in the healthy eating program is presented in Table 2. Examples from the HAPPY smartphone app are shown in Figure 2.

**Table 2:** Structure of weekly activities in the app.

Day	Content
Monday	Information and activity introduction
Tuesday	Recipe 1 and recipe 2
Wednesday	Short fact or advice 1
Thursday	Activity reminder: “How is it going?”
Friday	Recipe 3, recipe 4, and recipe 5
Saturday	Short fact or advice 2 and short fact or advice 3
Sunday	Activity evaluation: “How did it go?”

#### Download

At baseline, participants in the intervention group will download and be connected to the digital platform, where the study personnel (ie, trained nutritionists) can follow the individual progress of the user during the 12-week course on healthy eating habits in the app. Participants in the control group will be offered to download and be connected to the app at the 3-month follow-up. To avoid overlap with the 4-day food record, the app will be activated on the first Monday following the meeting with study personnel (ie, at baseline or the 3-month follow-up) and the intervention will thereafter follow for 12 consecutive weeks. An individual user account on the digital platform will be created for each participant by study personnel. Users will identify themselves in the app using a personal e-identification .

### Sample Size and Power Calculations

A total of 168 patients (84 per group) will provide 80% power at a 5% significance level to detect a clinically significant change of 4 mmol/mol in HbA_1c_ [18]. A standard deviation of 11.8 mmol/mol was estimated based on the average HbA_1c_ level (mean 53.4 mmol/mol, 95% CI 53.3-53.5) in Stockholm County in 2016 using data from the National Diabetes Register [19]. Based on earlier intervention studies within similar populations, a dropout rate of around 20% is expected. To cover for potential dropout, we will recruit a total of 200 patients (100 per group).

### Outcome Measures

#### Overview

A web-based questionnaire including assessment of lifestyle factors; a 4-day food record; clinical measurements of height, weight, waist circumference, blood pressure, and body composition; and blood sampling for measurement of HbA_1c_ and serum lipids will be given to all participants at baseline and at follow-up assessments at 3 and 6 months. At the 12-month follow-up, participants will respond to a final web-based questionnaire.

#### Dietary Intake From the 4-Day Food Record

Dietary intake is measured using a 4-day food record over 4 consecutive days, including at least one weekend day. Participants are given a paper diary and instructed by the study personnel, who are trained nutritionists, to write down everything they eat and drink during the period of recording. Type of meal (ie, breakfast, snack, lunch, dinner, etc) is also recorded. The estimated amount of food and beverages consumed can be recorded in different units, including number of items (eg, number of potatoes), weight (eg, 125 g chicken), or unit of volume (eg, 2 dL of milk or 1 cup of coffee). The participants are requested to maintain their usual diet during the days of recording. The software program Dietist Net (Kost och Näringsdata AB) is used by study nutritionists to calculate nutrient intake from the food records; the nutritionists also check the dietary recordings for completeness when they are returned. In the event of items being recorded in an unspecified way (eg, “fish” or “yogurt” without further specification), information on the most commonly consumed fish or yogurt is obtained from nationwide data [20] and entered into the nutrient calculations. Standard portion sizes available for each food item in the Dietist Net software are used if the amount of food was not specified.

The participants complete the food records, as well as respond to a food frequency questionnaire (FFQ), at baseline and follow-up after 3 and 6 months. While the food records will give detailed information on types of food items, portion sizes, and frequency and timing of intake during the day, data from the FFQs allow for comparison to other studies as well as to follow-up of study participants in this study after 12 months.

#### Body Composition

Weight, height, waist circumference, and body composition are measured by study personnel at baseline and at 3- and 6-month follow-ups. Weight is measured to the nearest 0.1 kg in light clothing without shoes, and height is measured to the nearest cm in a standing position. Waist circumference is measured around the waist, approximately 2 cm above the umbilicus, to the nearest cm. Body weight and waist circumference are measured once on each occasion. Body composition, including body fat and fat-free mass, is measured using a digital body composition analyzer, Model BC-418 (Tanita). The scale utilizes an 8-electrode bioelectrical impedance analysis with current going from foot to hand and from hand to foot on both sides of the body.

#### Blood Pressure

Blood pressure—systolic and diastolic—is measured by the study personnel using the M7 Intelli IT automatic electronic monitor with Bluetooth technology (Omron). Measurements are done with each participant in a seated position with legs uncrossed after the participant has been sitting for at least 5 minutes.

#### Biomarkers

Biomarkers are measured in fasting blood samples at baseline and at 3- and 6-month follow-ups. Study participants visit their closest primary care unit to have the blood samples taken. All samples are sent for analysis to the same lab connected to the Karolinska University Hospital in Stockholm, Sweden. HbA_1c_ (mmol/mol) is measured using the IFCC (International Federation for Clinical Chemistry and Laboratory Medicine) reference measurement procedure [21,22]. Triglycerides (mmol/L), total cholesterol (mmol/L), and high-density lipoprotein (HDL) cholesterol (mmol/L) are measured using the enzymatic method. Low-density lipoprotein (LDL) cholesterol (mmol/L) is calculated using the Friedewald equation:


LDL cholesterol = total cholesterol – HDL cholesterol – (0.45 × triglycerides)

The ratio of LDL cholesterol to HDL cholesterol is calculated from the levels of these lipoproteins.

#### Web-Based Questionnaire

Study participants respond to a web-based questionnaire at baseline and at follow-up after 3, 6, and 12 months. A link to the questionnaire is emailed to study participants on each occasion. A first reminder is sent if the participant has not responded within 1 week, and an additional reminder is sent 1 week after that if there is still no response. Each questionnaire takes approximately 30-45 minutes to complete and is comprised of several different sections as specified below. If not otherwise specified, the web-based questionnaire is comprised of the questions below.

*Background information* on marital status, education, and tobacco use (ie, smoking and snuff use) is assessed. Participants are also asked to report the year that they were diagnosed with type 2 diabetes; medication use related to hypertension, hyperlipidemia, and diabetes (ie, insulin or other treatment); and if they have changed their medication during the past 30 days. Background information is only collected from the baseline questionnaire.

*Dietary intake* is assessed using a 94-item semiquantitative FFQ. The FFQ has been validated previously in a random sample of Swedish men [23]. Spearman correlation coefficients for macronutrients comparing intake assessed using the FFQ, for an average of 14 24-hour recalls spread out over 1 year, were .44 (protein), .70 (total fat), .73 (carbohydrates), and .81 (alcohol). Participants report how often, on average, they consume each included food and beverage, including alcohol. An additional six questions developed by the Swedish National Food Agency, that are used in clinical practice, assessing overall dietary habits and risk use of alcohol are also included [24].

*Eating behavior* is assessed using the 21-item Three-Factor Eating Questionnaire [25]. The questionnaire includes questions related to cognitive restraint (six questions), uncontrolled eating (nine questions), and emotional eating (six questions).

*Physical activity* and *sedentary behavior* are assessed using general questions commonly used in clinical practice, regarding time spent doing physical activities at a moderate-intensity level or higher and sitting time [24], using the Active-Q questionnaire [26,27]. Active-Q includes 48 items and assesses habitual activity in four different domains: daily occupation, transportation, leisure time, and regular sports activities.

*Health-related quality of life* is assessed using the RAND-36, a questionnaire developed by the RAND Corporation [28]. RAND-36 comprises 36 questions within eight domains: physical functioning, role limitations due to physical health, role limitations due to emotional problems, energy and fatigue, emotional well-being, social functioning, pain, and general health. Responses from the individual domains are summarized into an overall physical component summary score and an overall mental component summary score. Additionally, a Swedish translation of the Life Engagement Test comprised of six questions is used to assess purpose in life [29].

*Sleeping habits* are assessed using a 13-item version of the Karolinska Sleep Questionnaire [30,31]. Participants report the time of going to bed the previous evening, time of waking up in the morning thereafter, and time from going to bed to falling asleep. They are also asked to report on sleep quality.

*Stress level* is assessed using a 14-item version of the Perceived Stress Scale developed by Cohen et al [32]. Participants are asked to respond to how often they perceive themselves to react in different, potentially stressful situations on a 5-point Likert scale, ranging from “Never” to “Very often.”

*Diabetes self-efficacy* and *distress* are assessed using the 20-item Swedish translation of the Problem Area in Diabetes Questionnaire [33]. Participants are asked to rate their distress with having diabetes on a 5-point Likert scale, ranging from “Not a problem” to “A serious problem.”

*Perceivedsocialsupport* for healthy eating habits is assessed by three questions regarding general support and support from friends and family. The questions were developed for this study and were based on the Physical Activity Social Support Scale [34]. Participants are asked to rate the statements “I experience support and encouragement to eat healthy food from *people around me,*” “...*my friends,*” and “...*my family*” using four response alternatives, ranging from “Agree completely” to “Do not agree at all.”

*Evaluation questions* about the usability of the app [35] and the contents of the healthy eating program are included in the 3-month follow-up questionnaire for participants in the intervention group and in the 6-month follow-up questionnaire for participants in the control group. This is done so that participants respond to the evaluation directly after having used the app. In total, users are asked to respond to 12 statements about the usability of the app (eg, “The app was easy to use”) and 12 statements regarding the content in the healthy eating program (eg, “The healthy eating program has made me reflect upon my dietary habits”) using a 5-point scale, ranging from “Do not agree at all” to “Completely agree.” In addition, users are also asked to leave a free-text comment regarding (1) what was good about the app and (2) what could be improved.

### Statistical Analysis

All data from clinical assessments, the questionnaires, and the app are anonymized and continuously stored at secure servers. Descriptive statistics will be summarized to describe participant characteristics at baseline and follow-up. Data will be checked for outliers and normality. Results will be stratified by control and intervention group. Baseline results will be tested to assess the success of the randomization in balancing characteristics. Differences between groups will be assessed using Student *t* tests, analyses of variance, and logistic regression. Any detected differences in baseline characteristics between the intervention and control groups will be taken into account as potential confounders in further analysis.

Testing for trends over time in outcomes will be conducted using statistical methods for longitudinal data. Generalized estimation equations will be used to assess the effect of both time and the intervention itself on outcomes. We will control for any unbalanced baseline characteristics. Testing for interactions will be conducted in the models, and intention-to-treat analysis will be performed to account for effects of crossover and dropout. Sensitivity analysis to account for missing data will be done. To further study if the effect of the intervention differs based on participant characteristics, stratified analysis will be performed.

Lastly, we will analyze user satisfaction and user statistics, including number and length of visits to the platform via the app. The number of completed actions during the active intervention will also be analyzed to assess user engagement with the app. Associations between use of the platform and characteristics of participants will be investigated.

## Results

The first study participants were recruited in January 2019. Data collection is ongoing. Recruitment is planned to continue until a total of 200 study participants have been included or up to the end of June 2021, whichever comes first. Data collection, including follow-up assessments, will be complete 1 year after recruitment of the last study participant. We expect to publish findings from the study by the end of 2021.

## Discussion

In the HAPPY trial, we aim to evaluate the effect of an mHealth intervention (ie, use of a smartphone app) promoting healthy eating habits in patients with type 2 diabetes. We know that a healthy diet is a key factor in the management of type 2 diabetes. However, implementing new habits (eg, healthier eating habits) and maintaining them is difficult. Nevertheless, interventions focusing on promoting healthier eating have been shown to lead to improvements in food habits [36]. Focusing on diet overall, instead of, for example, specific nutrients or energy intake, allows the individual to modify their existing dietary habits according to new advice, increasing the chance of successfully implementing new habits [37].

In a comprehensive review article by Ley et al [37], the authors concluded that the overall diet quality should be emphasized in dietary recommendations to patients. This was based on the gathered evidence showing that a higher intake of whole grains, fruits and vegetables, legumes, and nuts; moderate alcohol consumption; and a lower intake of refined grains, red and processed meats, and sugar-sweetened beverages were associated with a reduced risk of diabetes, as well as improved glycemic control and blood lipid levels in patients with type 2 diabetes. These results have also been supported in a later review [38]. The mHealth smartphone app evaluated in the HAPPY trial focuses on healthy eating habits in general, rather than the energy intake of specific nutrients.

Previous mobile- or app-based intervention studies targeting dietary intake or eating behavior have been summarized in two recent reviews, showing promising results [10,11]. Schoeppe et al [10] summarized results from studies using app strategies to target different lifestyle behaviors, including diet. Most apps targeting diet in adults were shown to be successful in improving dietary intake. Several of the included studies showed an increased fruit and/or vegetable intake or a decreased intake of sugar-sweetened beverages in the intervention groups compared to the control groups. The average duration of the intervention studies included in the review was 10 weeks (range 1-24). Further, Mandracchia et al [11] found that mHealth apps using self-monitoring were effective in increasing fruit and vegetable intake among adults or young adults with overweight. However, none of the apps targeting diet or eating behavior that were included in the two above reviews had been developed for, or evaluated among, patients with type 2 diabetes specifically.

In a study by Holmen et al [39], the authors showed good feasibility of using a mobile phone–based self-management system, including a dietary component, among patients with type 2 diabetes. After 1 year of using a mobile phone–based self-management system, between 30% and 40% of the individuals randomized to one of two groups using the system were substantial users of the app (eg, they had at least 50 interactions with the app during the past 6 months). Using an automated web-based program to support healthy diet has also been shown to improve dietary habits among this group of patients [12]. Further, a recent randomized controlled trial including patients with type 2 diabetes showed improvement in adherence to the Mediterranean diet and in diet quality overall after the use of a smartphone app over 3 months [13].

Noteworthy strengths of the HAPPY trial include the randomized design, a large sample size, objective assessment of outcomes (eg, measured clinical and anthropometric variables), and a priori calculation of statistical power. Further, the content of the HAPPY smartphone app was developed in collaboration with researchers, clinicians from primary care, and specialists from endocrinology clinics. The digital platform allows the patient and care provider to work together. This increases the chance of effectiveness, as interventions including both the health care system and patient have been found to be more effective than those targeting only one or the other [40]. Nevertheless, a limitation to our study is the lack of a pilot study testing feasibility and acceptability of the app among users. However, the healthy eating program is built into an existing digital solution that has been rigorously tested and is continuously updated to allow for the rapid development of iOS and Android systems. The stability of the digital solution is a strength, as users are less likely to stop using the app due to technical malfunctions. The use of various behavioral change techniques from different theoretical domains may also increase user engagement and decrease attrition in our study. Within the HAPPY trial, we are also collecting data on user engagement with, and usability of, the app and the healthy eating program.

A 4-day food record is used to assess dietary intake at baseline and follow-up. Although most dietary assessment methods are subjective and susceptible to social desirability, a food record has the advantages of being prospective (ie, does not rely on memory) and open ended with no limitation on what items can be reported. In the event of potential missing data in the food records (ie, unspecified food items being recorded or information on portion size is missing), this information will be added using data from a nationwide study of dietary intake in Sweden [20] or replaced by a standard portion size. This may affect results from the food records. However, such missing information is likely random. To enable long-term follow-up of dietary intake as well as comparison to other studies, participants also respond to an FFQ at baseline and follow-up after 3, 6, and 12 months.

Recruitment of study participants is performed at a number of different primary care centers located in areas with different populations and socioeconomic statuses. This increases the generalizability of our results to different groups of patients. However, the inclusion criteria of being able to communicate in Swedish and having access to a smartphone may be a limitation of the study. Type 2 diabetes is more common with increasing age. This could be a limitation, as knowledge of how to use a smartphone may be limited among older individuals, leading to a younger study population. Nevertheless, over 75% of individuals aged 66-75 years in Sweden use the internet on their phone [7]. While patients with limited knowledge of Swedish are more prevalent in areas of lower socioeconomic status, smartphone usage in Sweden is independent of socioeconomic status, and over 90% of all adults in Sweden own and use a smartphone [7]. The smartphone app was developed for usage on both Android and iOS devices, meaning that most smartphone users can download and use it.

To conclude, the app-based mHealth solution evaluated in the described randomized clinical trial has been developed taking into account the needs of patients, who request mHealth solutions to support a healthy lifestyle, as well as the health care system, which is in need of new, feasible solutions to meet the needs of increasing numbers of patients. Using mHealth strategies, real-time interaction with users is possible and health interventions can be delivered at any time. Further, focusing on diet quality, rather than on specific nutrients, food items, or energy intake, allows the user to apply new knowledge regarding healthy eating habits to his or her current eating patterns, as well as personal and cultural food preferences [41]. Such an individualized approach could be a key factor in promoting and supporting long-term adherence to healthier eating habits among persons with type 2 diabetes.

## Abbreviations

CONSORT: Consolidated Standards of Reporting Trials
CONSORT-EHEALTH: Consolidated Standards of Reporting Trials of Electronic and Mobile HEalth Applications and onLine TeleHealth
CVD: cardiovascular disease
FFQ: food frequency questionnaire
HAPPY: Healthy eating using APP technologY
HbA_1c_: glycated hemoglobin
HDL: high-density lipoprotein
IFCC: International Federation for Clinical Chemistry and Laboratory Medicine
LDL: low-density lipoprotein
mHealth: mobile health
SFO-V: Strategic Research Area Health Care Sciences
